# Soluble Urokinase Plasminogen Activator Receptor: A Useful Marker for Predicting Mortality in COVID-19 Patients

**DOI:** 10.7759/cureus.74438

**Published:** 2024-11-25

**Authors:** Tushar Sehgal, Ambika Anand, Gowtham Vijayabupathy, Maroof Khan

**Affiliations:** 1 Laboratory Medicine, All India Institute of Medical Sciences, New Delhi, New Delhi, IND; 2 Biostatistics, All India Institute of Medical Sciences, New Delhi, New Delhi, IND

**Keywords:** biomarker, covid-19, mortality, prognosis, soluble urokinase plasminogen activator receptor

## Abstract

Introduction

Efficient and practical healthcare based on prognostic indicators can reduce morbidity and mortality in hospitalized COVID-19 patients. Soluble urokinase plasminogen activator receptor (suPAR) predicts clinical outcomes and respiratory failure in SARS-CoV-2 patients, but additional research is needed. Among other characteristics, we aimed to evaluate the predictive value of suPAR in COVID-19 patients.

Methods

This observational study was conducted at the All India Institute of Medical Sciences in New Delhi between January and April 2022. Patients within the age range from 18 to 85 years with mild, moderate, or severe COVID-19 infections were included in the study. Twenty-one patients (group 1) had positive RT-PCR throat and nasal swabs. Nine patients (group 2) with fever but without COVID-19 were recruited as controls. Ninety patient samples were tested for suPAR on days 1, 5, and 10 utilizing suPARnostic AUTO Flex ELISA kits (ViroGates, Denmark).

Results

The median age was 59 years in both groups. COVID-19 was mild in six patients (29%), moderate in seven patients (33%), and severe in eight patients (38%). At a median follow-up of 10 days, 8 out of 21 patients (38%) in group 1 died, while none in group 2 died. Patients’ median suPAR levels were 4.35 ng/ml on day 1, 4.68 ng/ml on day 5, and 4.37 ng/ml on day 10. In the control group, suPAR levels were 1.6 ng/ml on day 1, 1.7 ng/ml on day 5, and 2.02 ng/ml on day 10. The suPAR levels were statistically significant on day 1 (p = 0.004), day 5 (p = 0.04), and day 10 (p = 0.007) between the patient and the control groups.

Conclusion

Patients who died had higher suPAR levels on days 1 (p = 0.007), 5 (p = 0.01), and 10 (p = 0.03) than survivors. The suPAR cut-off ≥ 3.64 (AUC = 0.82) predicts mortality with 88% sensitivity and 73% specificity.

## Introduction

In December 2019, several people in Wuhan City, Hubei Province, Central China, began to get pneumonia for no apparent reason [1]. Genome sequencing studies have shown that this pneumonia is caused by the coronavirus disease 2019 (COVID-19), which is also called the severe acute respiratory syndrome coronavirus 2 (SARS-CoV-2) [1-3]. Due to the global threat to health, the World Health Organization (WHO) has labeled COVID-19 a public health emergency of international concern [4]. Globally as of July 8, 2022, the virus has killed 6.34 million people and infected about 551 million others. United States, India, and Brazil are the most severely impacted nations [5]. The patients infected with SARS-CoV-2 exhibit a variety of clinical symptoms. It is well known that the majority have mild infections and that their symptoms are vague [6-8].

Fever, a dry cough, exhaustion, breathlessness, and myalgia are some of the typical presenting symptoms. It seems that symptoms like confusion, headaches, sore throats, rhinorrhea, chest pain, diarrhea, and vomiting are less frequent [6-8]. The patient may rapidly deteriorate with worsening signs of respiratory failure a week after infection [9]. Around 10% of patients were admitted to the intensive care unit (ICU) and given mechanical ventilation. Acute pneumonia, acute respiratory distress syndrome (ARDS), sepsis, and septic shock are severe disease manifestations that call for urgent ICU therapy [10]. In a large study in China on COVID-19 patients, sepsis was the most common complication in 59% of patients, followed by respiratory failure, ARDS, heart failure, and septic shock [11]. For all patients, sepsis manifested a median of nine days following the commencement of the illness [11]. The pathophysiology of sepsis in COVID-19 disorder needs to be better understood, although it was a frequent consequence that may have been caused directly by imbalanced host responses to SARS-CoV-2 infection [11,12].

The soluble urokinase plasminogen activator receptor, often known as suPAR, is a glycoprotein with a molecular weight between 55 and 60 kilodaltons [13]. suPAR is the soluble variant of urokinase plasminogen activator receptor (uPAR) that is secreted from the surface of a cell in response to inflammatory activation [13]. It has three different forms: I-III, II-III, and I. Researchers from Denmark discovered suPAR in the early 1990s and linked it to the growth of cancer [14]. suPAR may be found in blood, serum, urine, bronchoalveolar lavage, and cerebrospinal fluid, and its serum concentration remains stable under specific circadian and fasting settings [13]. As a plasminogen activator, suPAR enhances coagulation and fibrinolytic cascades and has a pro-inflammatory effect in many inflammatory illnesses [13]. In patients with sepsis, high levels of suPAR are associated with an increased risk of 30-day mortality [13]. Moreover, its overexpression significantly correlates with increased disease severity in ARDS patients. We aimed to evaluate the predictive value of suPAR in COVID-19 patients.

## Materials and methods

Study design

This is a cross-sectional study. The study was performed in the Department of Laboratory Medicine at the All India Institute of Medical Sciences, New Delhi, between January 2022 and April 2022. The institutional ethics committee approved the study (IEC-429/2020). This study was conducted as per the Declaration of Helsinki for medical research involving human subjects, including research on identifiable human material and data.

Participants

The inclusion criteria comprised patients aged between 18 and 85 years with mild/moderate/severe COVID-19 infections admitted to the hospital and were confirmed by a positive real-time polymerase chain reaction performed on the throat and nasal swabs [15]. Patients who came to the hospital for fever but were negative for COVID-19 throughout their entire hospital stay were included in the control group. All pediatric patients and those who did not provide consent were excluded.

Variables

The severity of COVID-19 was graded according to the Ministry of Health and Family Welfare (MOHFW) guidelines, India [15]. Mild disease was defined as asymptomatic patients or those with only upper respiratory tract symptoms and normal oxygen saturation on room air. Moderate COVID-19 was classified as patients with lower respiratory tract involvement, such as pneumonia, and oxygen saturation between 90%-94% on room air and/or a respiratory rate (RR) between 24-30/minute. Patients were classified as severe if the oxygen saturation was <90% on room air and/or a RR >30/minute, or if they had severe acute respiratory illness [16].

We recruited 30 patients, of which 21 (group 1) presented to the hospital with COVID-19-related symptoms, which were confirmed by a positive real-time polymerase chain reaction done on the throat and nasal swabs. Nine additional patients (group 2, control) were recruited who presented to the hospital with a fever but remained COVID-19-negative throughout their entire hospital stay. We tested 90 samples from 30 patients for suPAR using suPARnostic AUTO Flex ELISA kits (ViroGates, Denmark) at three different time points during the illness: day 1, day 5, and day 10.

suPAR testing

The blood samples were collected in the EDTA-anticoagulated tubes (BD Vacutainer® plastic tubes of 3 ml (buffered K2 EDTA 5.4 mg)) from the patients and the controls at three time points under all aseptic precautions by an experienced phlebotomist. The plasma (separated by gravity) was extracted using an automatic pipette, transferred to a microcentrifuge tube, and stored at -80°C in a deep freezer until tested. Once ready for testing, the samples were thawed entirely at room temperature. All samples were tested in duplicates as per the manufacturer's protocol.

Clinical and laboratory data

We collected the following data from the patients: clinical details, including comorbidities, COVID-19 severity, and laboratory parameters. Complete blood count (CBC) data, including hemoglobin, white blood cells (WBC), platelet count, absolute neutrophil count (ANC), absolute lymphocyte count (ALC), absolute monocyte count (AMC), absolute eosinophil count (AEC), absolute basophil count (ABC), neutrophil/lymphocyte ratio (NLR), neutrophil/monocyte ratio (NMR), and monocyte/lymphocyte ratio (MLR), were obtained from EDTA-anticoagulated blood samples (BD Vacutainer® plastic tubes of 3 ml (buffered K2 EDTA 5.4 mg)) analyzed using a Sysmex hematology analyzer (XN-9000) from Kobe, Japan. The results of serum creatinine (CREJ2, Roche Diagnostics, Rotkreuz, Switzerland​​​​​​​), serum urea (Ureal, Roche Diagnostics), total bilirubin (BILT3, Roche Diagnostics), serum ferritin (Elecsys®, Roche Diagnostics), and C-reactive protein (Tina-quant C-Reactive Protein IV, Roche Diagnostics) were obtained from serum samples collected in BD Vacutainer® SST™ tubes and analyzed on Cobas C701 (Roche Diagnostics) automated chemistry analyzer. There are no known potential sources of bias.

Statistical analysis

The data were summarized and analyzed using STATA software, version 14 (StataCorp LLC, College Station, TX) to describe the patient's characteristics. Qualitative data were reported as numbers and percentages. Quantitative data were expressed as mean ± standard deviation if the normality of the variable was assumed; otherwise, median (min, max). Data were tested for normality using the Kolmogorov-Smirnov test. A T-test was used to compare the values between the case and control groups if normality was established; otherwise, the Mann-Whitney U-test was performed to compare the non-normal variables. The Chi-square/Fisher's exact test was applied to set the categorical data's association. p-value < 0.05 will be considered statistically significant. suPAR as a biomarker is less well understood in COVID-19 patients; hence, we designed a pilot study. We recruited 21 patients and nine controls for this project.

## Results

A total of 30 patients were enrolled in the study. We tested 90 samples from 30 patients for suPAR using suPARnostic AUTO Flex ELISA kits (ViroGates, Denmark) at three different time points: day 1, day 5, and day 10. The characteristics of the cohort are presented in Table 1. Of the 30 patients included, the median age was 59 years (IQR: 24-85 years) in group 1, and the median age was 59 years (IQR: 29-65 years) in group 2. The male-to-female ratio was 2.5:1 in group 1 and 8:1 in group 2. The severity of COVID-19 was mild in six patients (29%), moderate in seven patients (33%), and severe in eight patients (38%). In group 1, eight patients (38%) had diabetes, and five (24%) had hypertension. There were no comorbidities in group 2. At a median follow-up of 10 days, eight out of 21 patients (38%) died in group 1, while none (0%) died in group 2.

**Table 1:** Characteristics of the cohort Group 1 represents the patient group, and group 2 represents the control group. The Chi-square or Fischer’s exact test was applied to set the categorical data’s association.

Characteristics	Group 1 (n = 21) (%)	Group 2 (n = 9) (%)	P-value
Age, years (IQR)	59 (24-85)	58 (29-65)	0.41
Gender (Male:Female)	2.5:1	8:1	0.39
Diabetes mellitus (%)	8/21 (38%)	0/9 (0%)	-
Hypertension (%)	5/21 (24%)	0/9 (0%)	-
COVID-19 severity			
Mild (%)	6/21 (29%)	-	-
Moderate (%)	7/21 (33%)	-	-
Severe (%)	8/21 (38%)	-	-
Mortality (%)	8/21 (38%)	0/9 (0%)	-

CBC parameters

The CBC results of the cohort are presented in Table 2. In the patient group, the mean hemoglobin values showed a decreasing trend on the three serial measurements on day 1 (11.09 ± 2.43 g/dL), day 5 (10.8 ± 2.73 g/dL), and day 10 (10.2 ± 2.69 g/dL). The patients' hemoglobin values were statistically significant on day 1 versus day 10 (p = 0.04). On the other hand, in the control group, the hemoglobin values remained similar on days 1, 5, and 10. The median WBC was higher in the patient group in comparison to controls on day 1 (p = 0.48), day 5 (p = 0.03), and day 10 (p = 0.01). The ANC was higher in the patient group in comparison to the control group on day 1 (p = 0.60), day 5 (p = 0.03), and day 10 (p = 0.004), while the ALC was lower in the patient group in comparison to the control group at day 1 (p = 0.73), day 5 (p = 0.04), and day 10 (p = 0.01). Moreover, in the patient group, serial lymphopenia was noticed on day 1 (0.7 (IQR: 0.5-2.1) x 10^9^/L), day 5 (0.6 (IQR: 0.1-1.5) x 10^9^/L), and day 10 (0.5 (IQR: 0.3-2.6) x 10^9^/L). This difference was statistically significant on day 1 versus day 5 (p = 0.001). The AMC in the patient group on day 1, day 5, and day 10 was 0.7 (IQR: 0.15-1.9) x 10^9^/L, 0.75 (IQR: 0.13-1.1) x 10^9^/L, and 0.68 (IQR: 0.01-1.3) x 10^9^/L. Both groups had similar AEC and ABC. In the patient group, the platelet count was 196 x 10^9^/L (IQR: 100-400) on day 1, 239 x 10^9^/L (IQR: 26-652) on day 5, and 211 x 10^9^/L (IQR: 85-600) on day 10. The difference between the platelets in the patient group and the control group was statistically significant on day 5 (p = 0.04).

**Table 2:** CBC parameters in the patient and the control groups Hb: Hemoglobin (normal range for females: 12-15 g/dl; normal range for males: 13-17 g/dl); ANC: Absolute neutrophil count (normal range: 2-7 × 10^3^/µl); ALC: Absolute lymphocyte count (normal range: 1-3 × 10^3^/µl); AMC: Absolute monocyte count (normal range: 0.2-1.0 × 10^3^/µl); AEC: Absolute eosinophil count (normal range: 0.02-0.5 × 10^3^/µl); ABC: Absolute basophil count (normal range: 0.02-0.1 × 10^3^/µl); NLR: Neutrophil/lymphocyte ratio (normal range: 0.78-3.53); NMR: Neutrophil/monocyte ratio (normal range: 0.37-2.87); MLR: Monocyte/lymphocyte ratio (normal range: 0.46-0.55); D1: Day 1; D5: Day 5; D10: Day 10. # The results of hemoglobin are in mean ± SD. All other results are in the median (interquartile range). T-test was used to compare the normal variables, while the Mann-Whitney U-test was performed to compare the non-normal variables.

Parameters (units), days	Group 1 (patients), n = 21				Group 2 (controls), n = 9				
	Values	P-value D1 vs D5	P-value D1 vs D10	Values		P-value D1 vs D5	P-value D1 vs D10	P-value (patient vs control), day	
#Hb (g/dL) D1	11.0 ± 2.43	0.27	0.04	10.4 ± 2.90		0.55	0.85	0.54	D1
Hb (g/dL) D5	10.8 ± 2.73			10.0 ± 3.48				0.40	D5
Hb (g/dL) D10	10.2 ± 2.69			10.5 ± 3.04				0.96	D10
WBC (× 10^9^/L) D1	14.6 (4.4, 22.2)	0.09	0.23	9.6 (5.7,29.9)		0.27	0.08	0.48	D1
WBC (× 10^9^/L) D5	16.3 (4.7, 32.4)			10.6 (5.4, 18.1)				0.03	D5
WBC (× 10^9^/L) D10	13.1 (3.9, 35.7)			9.0 (5.6, 15.7)				0.01	D10
ANC (× 10^9^/L) D1	12.9 (3.2, 20)	0.05	0.14	7.9 (3.9, 28.9)		0.18	0.07	0.60	D1
ANC (× 10^9^/L) D5	14.9 (3.2, 31.4)			7.8 (3.3, 17.4)				0.03	D5
ANC (× 10^9^/L) D10	12.0 (3.3, 33.9)			6.7 (3.2, 15.1)				0.004	D10
ALC (× 10^9^/L) D1	0.7 (0.5, 2.1)	0.001	0.12	0.9 (0.4, 1.3)		0.09	0.08	0.73	D1
ALC (× 10^9^/L) D5	0.6 (0.1, 1.5)			1.0 (0.4, 2.5)				0.04	D5
ALC (× 10^9^/L) D10	0.5 (0.3, 2.6)			1.1 (0.39, 1.8)				0.01	D10
AMC (× 10^9^/L) D1	0.7 (0.15, 1.9)			0.78 (0.16, 0.9)				0.76	D1
AMC (× 10^9^/L) D5	0.75 (0.13, 1.1)	0.73	0.47	0.56 (0.25, 1.4)		0.49	0.65	0.77	D5
AMC (× 10^9^/L) D10	0.68 (0.01, 1.3)			0.54 (0.16,1.2)				0.51	D10
AEC (× 10^9^/L) D1	0 (0, 0.4)	0.55	0.82	0 (0, 0.3)		0.08	0.39	0.02	D1
AEC (× 10^9^/L) D5	0 (0, 0.44)			0.1 (0, 0.2)				0.0006	D5
AEC (× 10^9^/L) D10	0 (0, 0.4)			0.1 (0, 0.2)				0.01	D10
ABC (× 10^9^/L) D1	0 (0, 0.1)	0.87	0.72	0 (0, 0.01)		0.85	0.95	0.08	D1
ABC (× 10^9^/L) D5	0 (0, 0.08)			0 (0, 0.05)				0.31	D5
ABC (× 10^9^/L) D10	0 (0, 0.07)			0 (0, 0.07)				0.23	D10
NLR D1	12.54 (2.7, 27)	0.0003	0.006	7.21 (4.04, 53.7)		0.11	0.07	0.54	D1
NLR D5	21.61 (3.5, 87.2)			5.55 (2.8, 43.7)				0.003	D5
NLR D10	19.79 (6.4, 80.9)			4.51 (2.9, 38.5)				0.0009	D10
NMR D1	18.01 (5.2, 42)	0.13	0.27	10.0 (4.4, 107.5)		0.18	0.28	0.63	D1
NMR D5	20.4 (5.35, 64.7)			13.3 (3.9, 68.7)				0.08	D5
NMR D10	20.6 (9.9, 95.0)			10.1 (3.5, 96.3)				0.03	D10
MLR D1	0.74 (0.21, 1.4)	0.002	0.08	0.73 (0.26, 1.05)		0.20	0.04	0.83	D1
MLR D5	1.11 (0.56, 2.9)			0.53 (0.36, 0.94)				0.0003	D5
MLR D10	1.01 (0.02, 2.5)			0.47 (0.14, 0.81)				0.02	D10
Platelet (× 10^9^/L) D1	196 (100, 400)	0.07	0.17	154 (90, 273)		0.88	0.28	0.19	D1
Platelet (× 10^9^/L) D5	239 (26, 652)			155 (120, 220)				0.04	D5
Platelet (× 10^9^/L) D10	211 (85, 600)			150 (53, 244)				0.05	D10

WBC ratios

We calculated the NLR, NMR, and MLR for both patients and controls (Table 2). In the patients, NLR was 12.54 (IQR: 2.7-27) on day 1, 21.61 (IQR: 3.5-87.2) on day 5, and 19.79 (IQR: 6.4-80.9) on day 10. NLR was statistically significant on day 1 versus day 5 (p = 0.0003) and day 1 versus day 10 (p = 0.006) in the patient group. The results were also statistically significant between patients and controls on day 5 (p = 0.003) and day 10 (p = 0.0009). In the patient group, NMR showed a rising trend; it was 18.01 (IQR: 5.2-42) on day 1, 20.4 (IQR: 5.35-64.7) on day 5, and 20.6 (IQR: 9.9-95.0) on day 10. The result was statistically significant between the patient and control groups on day 10 (p = 0.03). In the patient group, MLR was 0.74 (IQR: 0.21-1.4) on day 1, 1.11 (IQR: 0.56-2.9) on day 5, and 1.01 (IQR: 0.02-2.5) on day 10. The result was statistically significant between the two groups on day 5 (p = 0.0003) and day 10 (p = 0.02).

Biochemical parameters

The biochemical results (all values in median) of the cohort are shown in Table 3. In the patient group, the median serum urea levels were 51 mg/dL (IQR: 12-289) on day 1, 62.6 mg/dL (IQR: 18-166.7) on day 5, and 70.6 mg/dL (IQR: 18.2-213) on day 10. It showed a serial increase in the patient group in comparison to the control group on day 1 (p = 0.33), day 5 (p = 0.95), and day 10 (p = 0.70). Both groups did not have much variability in the median serum creatinine and total bilirubin levels. The median CRP levels in the patient group were 8.7 mg/L (IQR: 0.2-17.9) on day 1, 6.9 mg/L (IQR: 0.4-11.7) on day 5, and 1.07 mg/L (IQR: 0.9-4.6) on day 10. The results were not statistically significant in comparison to the control group on day 1 (p = 0.55), day 5 (p = 0.48), and day 10 (p = 1.0). The median serum ferritin levels in the patient group were 960 µg/L (IQR: 18.9-13638) on day 1, 872 µg/L (IQR: 186-2000) on day 5, and 1223.4 µg/L (IQR: 240-1589) on day 10. The results were not statistically significant in comparison to the control group on day 1 (p = 0.41), day 5 (p = 0.88), and day 10 (p = 0.25). In the patient group, the median suPAR levels were 4.35 ng/ml (IQR: 0.4-25.5) on day 1, 4.68 ng/ml (IQR: 0.4-30) on day 5, and 4.37 ng/ml (IQR: 0.53-24.31) on day 10. In the control group, the suPAR levels were 1.6 ng/ml (IQR: 0.7-3.2) on day 1, 1.7 ng/ml (IQR: 0-4.8) on day 5, and 2.02 ng/ml (IQR: 0-3.7) on day 10. The results were statistically significant in comparison to the control group on day 1 (p = 0.004), day 5 (p = 0.04), and day 10 (p = 0.007).

**Table 3:** Biochemical parameters in the patient and the control groups D1: Day 1; D5: Day 5; D10: Day 10. #All values are in the median (interquartile range). Urea (normal range: 17-49 mg/dl), creatinine (normal range: 0.7-1.2 mg/dl), total bilirubin (normal range: 0-1.2 mg/dl), CRP (normal range: 1.0-5.0 mg/L), ferritin (normal range in men: 30-400; women: 13-150 ng/ml), and suPAR (normal range: 2-3 ng/ml). T-test was used to compare the normal variables, while the Mann-Whitney U-test was performed to compare the non-normal variables.

Parameters# (units), days	Patients, n = 21			Controls, n = 9				
	Values	P-value D1 vs D5	P-value D1 vs D10	Values	P-value D1 vs D5	P-value D1 vs D10	P-value (patient vs control), day	
Urea (mg/dL) D	51 (12, 289)	0.97	0.44	54.4 (27.6, 183)	0.48	0.56	0.33	D1
Urea (mg/dL) D5	62.6 (18, 166.7)			49.6 (26.6, 162.1)			0.95	D5
Urea (mg/dL) D10	70.6 (18.2, 213)			53 (27.5, 224)			0.70	D10
Creatinine (mg/dL) D1	1.2 (0.4, 8.5)	0.28	0.40	1.5 (0.7, 6.9)	0.19	0.59	0.27	D1
Creatinine (mg/dL) D5	0.9 (0.4, 3.7)			1.9 (0.7, 2.9)			0.33	D5
Creatinine (mg/dL) D10	1 (0.45, 4.99)			1.68 (0.79, 5.8)			0.10	D10
Bilirubin (mg/dL) D1	0.8 (0.3, 1.3)	0.27	0.20	0.64 (0.3, 1.1)	0.75	0.89	0.30	D1
Bilirubin (mg/dL) D5	0.7 (0.3, 6.7)			0.56 (0.2, 1.4)			0.17	D5
Bilirubin (mg/dL) D10	0.9 (0.2, 11.9)			0.6 (0.27, 1.32)			0.17	D10
CRP (mg/L) D1	8.7 (0.2, 17.9)	0.93	0.21	9.9 (2.2, 20.3)	-	0.19	0.55	D1
CRP (mg/L) D5	6.9 (0.4, 11.7)			1.1 (1.1, 1.1)			0.48	D5
CRP (mg/L) D10	1.07 (0.9, 4.6)			2.6 (0.2, 5.9)			1.00	D10
Ferritin (µg/L) D1	960 (18.9, 13638)			699 (128, 1021)			0.41	D1
Ferritin (µg/L) D5	872 (186, 2000)	0.68	0.18	703 (618, 992)	0.59	0.65	0.88	D5
Ferritin (µg/L) D10	1223.4 (240, 1589)			370.3 (167, 573.7)			0.25	D10
suPAR(ng/ml) D1	4.35 (0.4, 25.5)	0.53	0.76	1.6 (0.7, 3.2)	0.35	0.21	0.004	D1
suPAR (ng/ml) D5	4.68 (0.4, 30)			1.7 (0, 4.8)			0.04	D5
suPAR (ng/ml) D10	4.37 (0.53, 24.31)			2.02 (0, 3.7)			0.007	D10

suPAR levels

At a median follow-up of 10 days, eight out of 21 patients (38%) died. All patients died due to ARDS and refractory septic shock due to COVID-19 disease in group 1, and no death occurred in group 2. The median suPAR levels in the patients who died were 5.71 ng/ml (IQR: 2.8-24.9) on day 1, 5.46 (IQR: 3.2-23.2) on day 5, and 5.01 (IQR: 2.4-24.3) on day 10. On the other hand, the median suPAR levels in the patients who survived were 1.9 ng/ml (IQR: 0.4-25.5) on day 1, 2.2 (IQR: 0-30.0) on day 5, and 3.0 (IQR: 0-24.0) on day 10.

**Figure 1:**
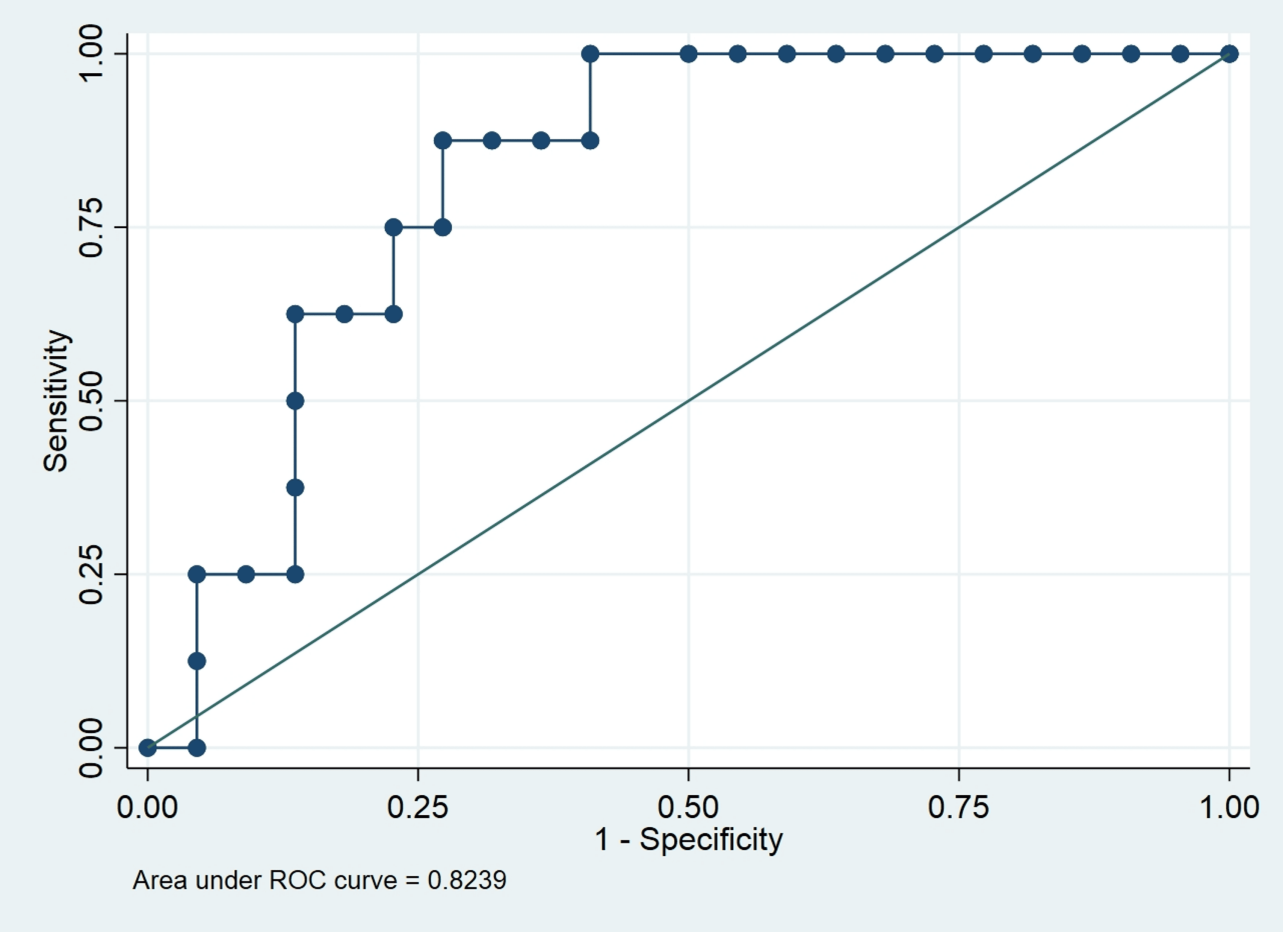
The receiver operating characteristic curve (ROC) showing that the suPAR cut-off ≥ 3.64 (AUC of 0.82) predicts mortality with a sensitivity of 88% and specificity of 73%

The receiver operating characteristic curve (ROC) curve showed a suPAR cut-off ≥ 3.64 (AUC of 0.82), predicting mortality with a sensitivity of 88% and specificity of 73% (Figure 1). Those patients who died had higher levels of suPAR in comparison to those who survived at all three time points. The difference was statistically significant with p = 0.007, p = 0.01, and p = 0.03 on day 1, day 5, and day 10, respectively (Table 4).

**Table 4:** Comparison of suPAR levels# among those who died and survived #All results are in median (interquartile range; ng/ml). A T-test was used to compare the normal variables.

Day	Dead, n = 8/30	Alive, n = 22/30	P-value
D1	5.71 (2.8, 24.9)	1.9 (0.4, 25.5)	0.007
D5	5.46 (3.2, 23.2)	2.2 (0, 30.0)	0.01
D10	5.01 (2.4, 24.3)	3.0 (0, 24.0)	0.03

## Discussion

The SARS-CoV-2 has the potential to cause COVID-19, which ranges from asymptomatic to severe. Patients must be hospitalized if they require oxygen or have multiple organ dysfunction during the first week of the disease because pneumonia with eventual respiratory failure can arise during this time [11,17]. At this stage of the infection, it is difficult to predict which patients will require intensive oxygen supplementation, such as high-flow nasal oxygen treatment, continuous positive airway pressure therapy, or mechanical ventilation, and how long they will be in the hospital [17]. People at risk of getting severe COVID-19 need to be identified using prognostic markers as soon as feasible so that appropriate care can be delivered either preventively or at the earliest possible stage of the disease [17]. Counts of lymphocytes, neutrophils, platelets, NMR, NLR, D-dimer, interleukin-6, CRP levels, and lactate dehydrogenase (LDH) levels may be able to help differentiate between severe and non-severe COVID-19 [11,18,19].

suPAR as a biomarker of inflammation is not unique to any cell type and can be tested in plasma or serum [20]. The measurement results do not alter throughout the day [20]. suPAR levels are increased not only by certain diseases but also by their severity. Patients with diabetes mellitus, cardiovascular disease, and chronic obstructive pulmonary disease might benefit from using suPAR as a predictive tool [20]. Rasmussen et al. reviewed 4343 consecutively admitted patients from the acute medical unit at a large Danish university hospital [21]. They found that suPAR is strongly associated with disease severity, readmission, and mortality, which indicates that suPAR adds information to established prognostic indicators. In addition, they found that suPAR is strongly associated with a shorter length of stay in the hospital. Patients with low levels of suPAR have a lower risk of mortality and readmission, whereas patients with high levels of suPAR have a higher risk of experiencing adverse events [21].

Santeri et al. evaluated 1747 patients with acute medical conditions at the emergency department (ED). The cut-offs of suPAR were defined for risk classification for patients seeking care at the ED [20]. Patients with acute medical conditions with a low, medium, or high risk of 30- and 90-day mortality can be identified using suPAR cut-offs of below 4, between 4 and 6, and above 6 ng/ml, respectively [20]. Rasmussen et al. conducted another study on 17312 patients with acute medical conditions who were treated in ED [22]. The researchers concluded that the addition of suPAR to the National Early Warning Score significantly improved the prediction in low-risk and high-risk acute medical patients. In addition to this, it was discovered that a suPAR level of <3 ng/ml is related to a low risk of readmission and mortality; a level of 3-6 ng/ml is associated with a medium risk; and a level of more than 6 ng/ml is associated with a high chance that requires therapeutic attention [22]. Rovina et al. in their study on 57 COVID-19 patients concluded that an elevated suPAR level of >6 ng/mL is a robust predictor of the requirement of mechanical ventilation with a positive predictive value and negative predictive value of 85.7% and 91.7%, respectively [23]. Huang et al. demonstrated that suPAR levels in 117 COVID-19 patients were significantly higher than the levels in healthy controls (5.51 ± 2.53 ng/mL vs 1.97 ± 0.78 ng/mL, p < 0.0001). They concluded that suPAR levels increase as the disease worsens [24]. In another observational study by Altintas et al. on 386 COVID-19 patients in Denmark, the researchers concluded that patients with suPAR levels of 4 or 6 ng/mL had a low or high probability of developing the infection, respectively [25]. Patients with suPAR levels of ≥6 ng/mL required mechanical ventilation or had increased mortality [25].

In our study of 30 COVID-19 patients enrolled at three time points viz day 1, day 5, and day 10, the median age was 59 years in the patient group. The severity of COVID-19 was mild in six patients (29%), moderate in seven patients (33%), and severe in eight patients (38%). At a median follow-up of 10 days, eight out of 21 patients (38%) died. All patients died due to ARDS and refractory septic shock complicated by COVID-19 disease. Those patients who died had higher levels of suPAR on day 1, day 5, and day 10 than those who survived. The difference was statistically significant with p = 0.007, p = 0.01, and p = 0.03 on day 1, day 5, and day 10, respectively. The ROC curve shows that the suPAR cut-off ≥ 3.64 (AUC of 0.82) predicts mortality with a sensitivity of 88% and specificity of 73%.

Data are scarce on suPAR in COVID-19 patients from Africa and India, with only two studies reported from India. Chandna et al. recruited 426 adults with moderate COVID-19 infection from two hospitals in India to develop and validate a model for supplemental oxygen requirement [26]. They concluded that the model containing NLR, suPAR, or IL-6 displayed promising discrimination (c-statistics ranging from 0.72 to 0.74) and calibration and provided more value than a model that included the clinical parameters alone [26]. In a separate piece of research conducted by Sarif et al., the plasma suPAR level was connected to a unique plasma proteome associated with coagulation abnormalities and complement activation [27]. The ROC analysis determined that the suPAR cut-off value >1.99 ng/ml predicts mortality [27].

Strengths and limitations

Our findings demonstrated that suPAR levels were greater and statistically significant on days 1, 5, and 10 in patients with COVID-19 who succumbed compared to those who survived. ROC analysis demonstrated that a suPAR cut-off ≥ 3.64 (AOC of 0.82) accurately predicts mortality with a sensitivity of 88% and a specificity of 73%. In the future, we may plan a study on a large scale with automated testing of suPAR in emergency settings to help triage patients for better and early disease management.

Our study had a few shortcomings, the most notable of which were the follow-up and the relatively small sample size. To evaluate the importance of suPAR in COVID-19 patients, a large sample size and a more extensive follow-up period are required. To determine whether this inflammatory marker is significant, we need to do additional research using our populations of COVID-19 patients.

## Conclusions

Our findings demonstrated that suPAR levels were greater on day 1 (p = 0.007), day 5 (p = 0.01), and day 10 (p = 0.03) in patients with COVID-19 who succumbed compared to those who survived. ROC analysis demonstrated that a suPAR cut-off of ≥3.64 (AOC of 0.82) accurately predicts mortality with a sensitivity of 88% and a specificity of 73%.
